# Egg-Laying “Intermorphs” in the Ant *Crematogaster smithi* neither Affect Sexual Production nor Male Parentage

**DOI:** 10.1371/journal.pone.0075278

**Published:** 2013-10-10

**Authors:** Jan Oettler, Michiel B. Dijkstra, Jürgen Heinze

**Affiliations:** 1 Biologie I, Universität Regensburg, Regensburg, Germany; 2 Department of Ecology and Evolution, University of Lausanne, Lausanne, Switzerland; Stanford University, United States of America

## Abstract

We study male parentage and between-colony variation in sex allocation and sexual production in the desert ant *Crematogaster smithi*, which usually has only one singly-mated queen per nest. Colonies of this species are known to temporarily store nutrients in the large fat body of intermorphs, a specialized female caste intermediate in morphology between queens and workers. Intermorphs repackage at least part of this fat into consumable but viable male-destined eggs. If these eggs sometimes develop instead of being eaten, intermorphs will be reproductive competitors of the queen but—due to relatedness asymmetries—allies of their sister worker. Using genetic markers we found a considerable proportion of non-queen sons in some, but not all, colonies. Even though intermorphs produce ∼1.7× more eggs than workers, their share in the parentage of adult males is estimated to be negligible due to their small number compared to workers. Furthermore, neither colony-level sex allocation nor overall sexual production was correlated with intermorph occurrence or number. We conclude that intermorph-laid eggs typically do not survive and that the storage of nutrients and their redistribution as eggs by intermorphs is effectively altruistic.

## Introduction

As in non-social organisms, resource availability constrains how much colonies of wasps, bees, and ants (order Hymenoptera, with haplodiploid sex determination) can allocate to reproduction: the greater the number and size of dispersing virgin queens and males raised per reproductive season, the less workers can be raised to sustain colony growth. It is thus imperative that workers, who nurse the brood, raise only those sexuals that yield the highest fitness return on investment.

The discrimination by workers in favor of some brood categories is the expression of two potential queen-worker conflicts [1]. The first conflict, over which males should be raised, occurs because all females (e.g. workers, mother queens, mature virgin queens) can lay unfertilized, haploid, male eggs and thus compete over the production of males (for exceptions see [2], [3]). It has been shown that workers and queens of many ants, bees, and wasps selectively eat those eggs to which they are least related (“worker policing”, “selfish policing”, or “queen policing”, depending on whether the egg-eater is a non-reproductive or reproductive worker or a queen: [4], [5]).

The second conflict is over the sex ratio: queens are equally related to daughters and sons, so that an average colony sex allocation ratio of 0.5 (i.e. 50% energy invested in virgin queens and 50% in males) is typically optimal for queens (for exceptions see [6], [7]). But due to haplodiploidy, workers are more related to sisters than to brothers, so that the workers' optimum for the average colony sex allocation ratio is always more virgin queen-biased than the queen's optimum [6]–[10]. A colony's final sex ratio is constrained by the queen's output of female (fertilized) and male (unfertilized) eggs, and other females' output of exclusively male eggs [7], [9]–[16], but nurse workers are known to influence the final sex ratio by preferential cannibalization of larvae [17]. The queen-worker conflict over the sex ratio has been “won” by the workers in many taxa (reviewed in [18]), but there are exceptions to this general pattern [13], [14]–[16].

Queen and worker optima can predict not only the average colony sex allocation ratio, but also the between-colony variation in sex ratio. A non-unimodal distribution of colony sex ratios may indicate that workers follow a conditional behavioral strategy for sex allocation [11], [19]–[21]. For example, workers of many species have been shown to increase their inclusive fitness by preferentially raising virgin queens or males, depending on whether the colony-specific ratio of the relatedness of workers to sisters versus brothers is smaller versus greater, respectively, than the population average (reviewed in [22]).

Another well-documented conditional strategy of eusocial Hymenoptera is to raise preferentially virgin queens in large, well-fed colonies and preferentially males in small, poorly fed colonies [20], [23]–[31]. Food-dependent sex ratios are an example of the Trivers-Willard theory of sex allocation, which predicts that females (or colonies in eusocial taxa) with few resources should preferentially produce offspring of the sex whose reproductive success is least sensitive to variation in maternal investment ([32]–[35]; reviewed in [36]). Furthermore, the cost ratio hypothesis ([20], but see [37]), the constant male hypothesis [38] and the multifaceted parental investment theory [39] predict sex allocation based on resource availability. In eusocial Hymenoptera, food limitation is thought to impinge most on the virgin queens' future reproductive success because: (1) males are smaller, live considerably shorter, and have smaller fat reserves than queens [2], [20], [29], [40]; (2) males of ants exclusively produce sperm as pupae, so their fecundity and fertility is hardly affected by food availability after eclosion [40]; (3) males do not fight and hence should benefit less from a larger body size than queens, who are known to fight over nest sites during the colony founding stage in many taxa ([40]–[44]; for exceptions to points 2–3 see [45]–[47]).

Here we study an unusual extension of the typical queen-worker conflict over sex allocation and male parentage in the ant *Crematogaster smithi* from Arizona. In addition to workers and queens, colonies may contain one or several “intermorphs,” which are intermediate between queens and workers in body size, morphology, and ovarian morphology [48], [49]. As such they have to be considered different from major worker castes with specialized morphological adaptations to distinct tasks. Major worker castes occur in many ant lineages. The functions of major workers are as diverse as food processing in *Pheidole*, nest entrance blocking in turtle ants, soldier behavior in army ants or leaf carrying in leafcutter ants [50]. Intermorphs are widespread in the subgenus *Crematogaster* (*Orthocrema*) (e. g., *C.* cf. *baduvi*, *C. curvispinosa*, *C. bryophilia*, *C. nigropilosa*, *C. rasoherinae* [51]–[54]). In *C. smithi*, intermorphs lack a spermatheca for the storage of sperm but have developed ovaries with numerous developing oocytes. The behavior of *C. smithi* intermorphs [48] shows that they are clearly not a soldier caste in spite of having been described as one recently [54]. Most of the eggs laid by intermorphs are eaten by the larvae and by the queen, though a previous, small-scale genetic study indicated that at least some survive and develop into adult males [55].

The aim of the present study was to determine who benefits from the intermorphs' presence: the whole colony, the queen, the collective of the workers, or only the intermorphs themselves. Like “fat body repletes” (*cf.* [56]) known from other ants, intermorphs might serve as sinks for food gathered by workers and store energy and nutrients as fat, which they can redistribute to colony mates in the form of consumable eggs [48], [49]. As the availability of food strongly affects sex allocation ratios, the presence of intermorphs might be associated with the production of more sexuals and a more female-biased sex allocation ratio. Alternatively, intermorphs might be reproductive allies of their worker sisters. *C. smithi* queens are singly mated [55] and workers therefore are more closely related to intermorph sons than to queen sons. To increase relatedness to the male brood while avoiding the expected colony-level productivity costs of having numerous reproductively inefficient egg-laying workers in the colony (e.g. [57]–[59]), workers could thus benefit from raising a few fecund, reproductively efficient intermorph sisters whose sons will replace queen sons (see also [60] for male-producing intermorphs in *Polyergus samurai*).

We estimated sexual production, relative sex ratio bias towards costly virgin queens, and male parentage (and thus the average relatedness of intermorphs, queens, and workers to the males) in field and laboratory colonies of *C. smithi* with varying numbers of intermorphs. We also collected life history data (colony composition, egg laying rates, worker-worker relatedness and effective queen mating frequency estimated with genetic markers) from a considerably larger number of colonies compared to previous studies [48], [49], [55].

## Methods

### Study species

*Crematogaster smithi* (Creighton 1950) occurs in mixed forests in the arid mountains of Arizona, USA, and Sonora, Mexico, and lives in underground nests consisting of a single tunnel (up to 40 cm deep) with up to 10 nest chambers. Between July and early August, multiple synchronized mating flights occur during the evenings of rainy days. Queens found new colonies independently and claustrally, i.e. they start a new nest without the help from workers and raise the first brood without foraging. Sexuals develop from eggs laid in late summer, overwinter as larvae, pupate around March and reach maturity around June. During the cool evenings and nights, forager workers hunt for small insects and consume these outside the nest (J.O. & C.R. Smith, personal field observations).

In queenright colonies, queens, workers, and intermorphs lay viable “large” eggs (∼0.5 mm long) and in addition also flaccid “small” eggs (<0.4 mm long) that fail to develop. Queens produce proportionally more (∼19% of queen-laid eggs) small eggs than intermorphs (∼2%) and workers (∼3) [48] Queens and larvae exclusively feed on these “large” and “small” eggs, but it is unknown whether they discriminate between worker-, intermorph-, and queen-laid eggs.

### Collection and maintenance of colonies

In July 2005 and 2006 we excavated complete colonies of *C. smithi* near the Southwestern Research Station (SWRS) in the Chiricahua Mountains, Arizona. A permit was obtained from the SWRS to work on Forest Service property. Immediately after collection, we counted all workers, intermorphs, mother queens, virgin queens, and males, and preserved all virgin females and males and a sample of workers in >99% ethanol for genotyping. Adult sexuals, worker larvae and pupae, and callow (i.e. recently emerged from the pupa) and mature workers, but no sexual larvae or pupae were present at the time of collection. Colonies were transported to Germany and maintained in the laboratory (24°C and 60% relative humidity, with a day/night cycle that was twice a month adjusted to Arizona's latitude), where they were fed three times weekly with chopped cockroaches and diluted honey. In June 2006, we collected newly produced adult males from five of these 2005 laboratory colonies containing intermorphs – all of which had retained their queen – and stored them in >99% ethanol for additional genotyping. In February 2009, we sampled additional males from four colonies that had been maintained in the laboratory since 2005 and which did not contain any intermorphs. We also analyzed caste composition data from additional 74 colonies collected between 1992 and 1998 [48], [49], [55] from the same population for which no sex allocation data were available. For an overview of the data used see Table 1.

**Table 1:** Summary table of samples.

Collection years	Nr colonies	Used for: Caste composition	Used for: Sex allocation (field)	Used for: Male parentage (field)	Used for: Male parentage (lab)
**1992–1998**	74	74			4 colonies with intermorph (45 males [median 10.5, range 7–17], 46 workers); re-analyzed from [55]
**2005**	38	38	38	9 colonies (149 workers, 156 males, colonies with intermorph), Different colonies than for Male parentage in lab colonies	
**2006**	18	18	18		5 colonies with intermorph (60 males [median 13, range 6–14]). 4 colonies without intermorph (38 males [median 11.5, range 8–15], 40 workers)

### Microsatellite genotyping of workers and males

To estimate (1) the proportion of non-queen sons (i.e. sons of workers or intermorphs) and (2) the average mating frequency of queens, we genotyped a total of 149 workers (range 12–19 per colony) and 156 males (range 3–28 per colony) from nine “queenright” (i.e. containing a mother queen) field colonies collected in 2005 containing (1–6) intermorphs. Between 2006–2009, we genotyped 60 males from five queenright laboratory colonies containing (1–7) intermorphs and 38 males and 40 workers from four colonies without intermorphs. We also included data on 45 males and 46 workers from four colonies from an earlier study [55]. We extracted genomic DNA with the Gentra Puregene Kit (Qiagen, Hilden, Germany) and amplified the two microsatellite loci C20 (GenBank accession number AF 167066) and C9 (AF 167064) with the protocols in [50]. We ran PCR products on an ABI 310 (Applied Biosystems, Foster City, CA) and manually scored peaks. Both loci were variable (C20: 4 alleles, expected heterozygosity *H_e_* = 0.62; C9: 10 alleles, *H_e_* = 0.82) [55]. We estimated the population-wide allele frequencies and the mean within-colony relatedness with the program Relatedness (version 4.2; [61]). We deduced the most likely genotype of the single queen per colony and her mate(s) with the program Matesoft Silver (version 1.0; [62]). We estimated the effective mating frequency of queens 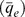 with equation 2 in [63], which corrects for unequal contributions of multiple fathers to the female offspring.

### Correction for imperfect genetic detection of non-queen sons

“Non-queen sons”, i.e. sons of intermorphs or workers, can only be “detected”, that is genetically distinguished from queen sons if they have inherited an allele from their maternal grandfather that their maternal grandmother did not carry. We estimated the probability *P_d,i_* of detecting a non-queen son in each colony *i* with the standard formula 2 in [64], which assumes that both of our microsatellite loci are unlinked. We were interested in 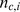, the true number of non-queen sons in each colony *i*. To estimate 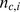, two sources of binomially distributed errors must be taken into account: (1) the numbers of non-queen sons in the sample of genotyped males that are detectable versus non-detectable, assuming fair meiosis and no effect of microsatellite genotype on survival of males; and (2) the numbers of queen and non-queen sons that we haphazardly sampled for genotyping from among all the males produced by the colony. To estimate 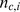 and its statistical uncertainty, we wrote a script (available upon request from the second author) in the “R” language (http://cran.r-project.org).

Our script returns a vector of likelihoods 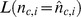, for 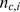, calculated with (Eqn. 1):
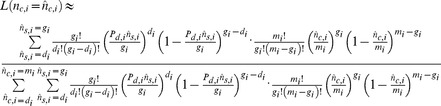
in which 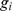 is the known number of genotyped males from colony *i*; 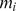 is the known total number of males produced by colony *i*; 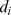 is the number of detectable non-queen sons in the genotyped sample from colony *i*; 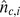 is any theoretically possible value for the unknown true value of 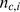 (see above) and gets sequentially set at 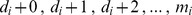; and 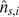 is any theoretically possible value for the unknown true value of 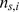 (the total number of non-queen sons in the genotyped sample from colony *i*, both detectable and non-detectable) and gets sequentially set at 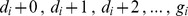. The vector 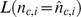 has a unimodal shape, similar to a binomial density function but with a higher kurtosis. The value of 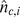 for which 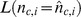 is maximal is the maximum likelihood estimate for 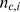. The lower 95% confidence limit of this estimate is the smallest value of 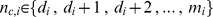 for which the following inequality is true:
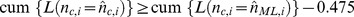
(2)in which 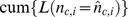
is the cumulative distribution function of 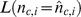 and 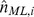 is the maximum likelihood estimate for 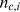. Analogously, the upper 95% confidence limit for the estimate for 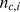 is the highest value of 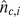 for which the following inequality is true:
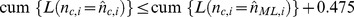
(3)To verify that our script returns accurate values for the maximum likelihood estimate of 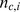 and its 95% confidence limits, we used a large simulated data set of 18450 colonies, with ranges of possible values of 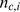, 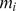, 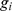, and *P_d,i_* and drawing values from the stochastic variables *n_s,j_* and *d_i_* from an uniform distribution. Indeed, we found that the accuracy (i.e. the estimated value 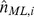 divided by the *a priori* known true value 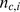) of the maximum likelihood estimate was 1.04±0.004 (mean ± SE; N = 18450 simulated colonies), which is close to the expected value of 1 for perfect accuracy. Similarly, the proportion of *a priori* known true values of 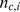 that lay between the obtained 95% confidence limits was 0.93, which is close to the expected value of 0.95 for perfect accuracy. Equations 1–3 and our script thus give reliable results.

### Reproductive success of workers and intermorphs

Our microsatellite markers could distinguish between queen and non-queen sons, but not between intermorph and worker sons. We therefore estimated the relative contributions of intermorphs and workers as follows. If intermorphs have sons that are raised at the expense of queen sons, the proportion of non-queen sons among all the males in the colony (*n*_c,i_, for which 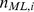 is the maximum likelihood estimate should increase with increasing numbers of intermorphs per colony. We tested this prediction by fitting a generalized linear model (GLM) with a “logit” link function and pseudo-binomial errors (i.e. correcting for overdispersion of the response with an empirical scale factor) with the proportions 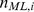 as response variable, “Laboratory or field colony?” (N = 12 and N = 7, respectively) as fixed factors, “Square root of number of intermorphs per colony” (median after transformation: 1.2; range: 0–2.6) as covariate, and the total number of males per colony as weights. The vertical intercept thus yields an estimate for the workers' joint contribution to the adult males in the absence of intermorphs, while the slope estimates the average reproductive success per intermorph. The covariate had been square-root-transformed to avoid giving excessive weight to the colonies with the largest number of intermorphs in the genotyped sample. “Colony” could not be made a random factor, because each data point consisted of a single unique colony. We sequentially removed the least significant term in each step, until only significant terms remained (see [21] for details).

As an independent estimate of the relative contributions of intermorphs and workers to the production of non-queen sons, in 2006 we compared the egg laying rates of intermorphs and workers in a bioassay described in [48]. Briefly, we isolated broodless colony fragments from laboratory colonies consisting either of ten workers and one intermorph (3.3 ± SD 1.5 replicates for each of seven colonies), or 11 nest mate workers (2.1 ± SD 1.4 replicates for each of 18 colonies). For comparison with queen fecundity we also included colonies consisting of one queen and 10 workers (one replicate for each of 15 colonies). We counted the number of eggs present at 6, 12, and 18 d after the females' separation from the colony, without distinguishing between “small” (∼3%) and “large” (∼97%) eggs. From these egg-laying data we estimated the proportion of males that workers and intermorphs could theoretically produce based on the estimate for 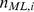.

### Do intermorphs affect sexual production and sex ratio?

To determine whether colonies with intermorphs are more productive than colonies without intermorphs, we estimated sexual productivity in field colonies (July 2005 & 2006; see above) with (cf. [21], [65]):
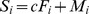
in which S_i_ is the “Sexual production” of colony i (a relative measure of energy allocated to sexuals, expressed in male equivalents), c (the “cost ratio”) is a correction factor for the greater colony-level productivity and maintenance cost of virgin queens compared to males, and F_i_ and M_i_ are the number of virgin queens and males (only mature sexuals were present in the colonies) in colony i on the day of excavation, respectively.

We estimated c with (cf. [21], [37], [66], [67]):
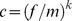
in which *f* and *m* are the average dry masses of mature virgin queens and males, respectively, and *k* is the “metabolic parameter” that corrects for the lower respiration rate and higher fat content of ant virgin queens compared to males [67]. We used the across-species average value *k* = 0.7 [66], [67] that is typically used in sex ratio studies on ants. To estimate *f* and *m*, we dried flight-ready sexuals from the field in 2005 that had been preserved in >99% ethanol for ≥72 h at 50–55°C, and weighed them to the nearest 0.01 mg.

To determine whether the number of intermorphs affects sexual production or biases the sex ratio towards virgin queens (the sex for which average fitness is thought to increase fastest with increasing nutrition), we performed a GLM on the colony-level numerical sex ratios (i.e. the proportion of virgin queens among all the sexuals in the colony) with quasi-binomial errors. We tested the covariates “Square root of number of intermorphs per colony,” “Number of workers” and their second order interaction term, the covariate “Sexual production *S_i_*”, and the factor “Year”, and stepwise removed the least significant term.

## Results

Information about the caste composition (38 colonies from 2005 and 18 colonies from 2006) and genetic structure of the colonies is summarized in Table 2, which also contains data from 74 colonies that had been collected between 1992 and 1998 at the same site [48], [49], [55]. Sex ratio data for the latter colonies were unavailable because they had been collected outside the mating season. There was a significant positive association between the intermorph number and worker number across all colonies (Spearman correlation coefficient *r_s_* = 0.384, n = 115, p<0.007).

**Table 2:** Summary of colony parameters are given as mean ± SE (range) [number of colonies].

Estimate	All field colonies (1992–2006)	Reproductive field colonies (2005/2006)
Dry mass (mg) of mature virgin queens 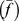	–	1.41±0.07 [7]
Dry mass (mg) of mature males 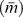	–	0.09±0.003 [107^1^]
Virgin queen-to-male energy cost ratio (*c*)	–	6.97±0.81 (SD^2^)
Queens per colony	1±0.012 (1–2) [130]	1 [31]
Percentage of colonies with two queens	1.5% [130]	0% [31]
Intermorphs per colony	1.8±0.22 (0–16) [130]	2.97±0.68 (0–16) [31]
Percentage of colonies without intermorphs	29% [130]	32.26% [31]
Workers per colony	305±22 (4–1370) [130]	538±56 (117–1370) [31]
Effective queen mating frequency 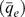	–	1.14±0.12 (1–2) [9]
Probability of detecting non-queen sons 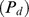	–	0.40±0.05 (0–0.50) [13^3^]
Regression relatedness among workers	–	0.73±0.06 [9]
Regression relatedness among males	–	0.52±0.12 [9]

1 Collected from a mating flight in 2005.

2 Only a single estimate is available for this parameter because it has been derived from other parameters.

3 Also includes four reproductive laboratory colonies.

The relatedness among nest mate workers in reproductive field colonies was r = 0.73±0.06, which does not significantly differ from the value of 0.75 expected for colonies with a single, singly mated queen (t_8_ = 0.33, p = 0.75). However, the average effective queen mating frequency was slightly above 1 (*q_e_* = 1.14±0.12) because a second queen's mate was detected in two (22%) of the nine genotyped sexual-producing field colonies. In another two colonies the queen and her mate did not have informative alleles at both loci and thus the probability *P_d_* of detecting a non-queen son was zero. These two colonies were discarded from the subsequent analysis because a maximum likelihood estimate could not be calculated.

### Male parentage

**Figure 1:**
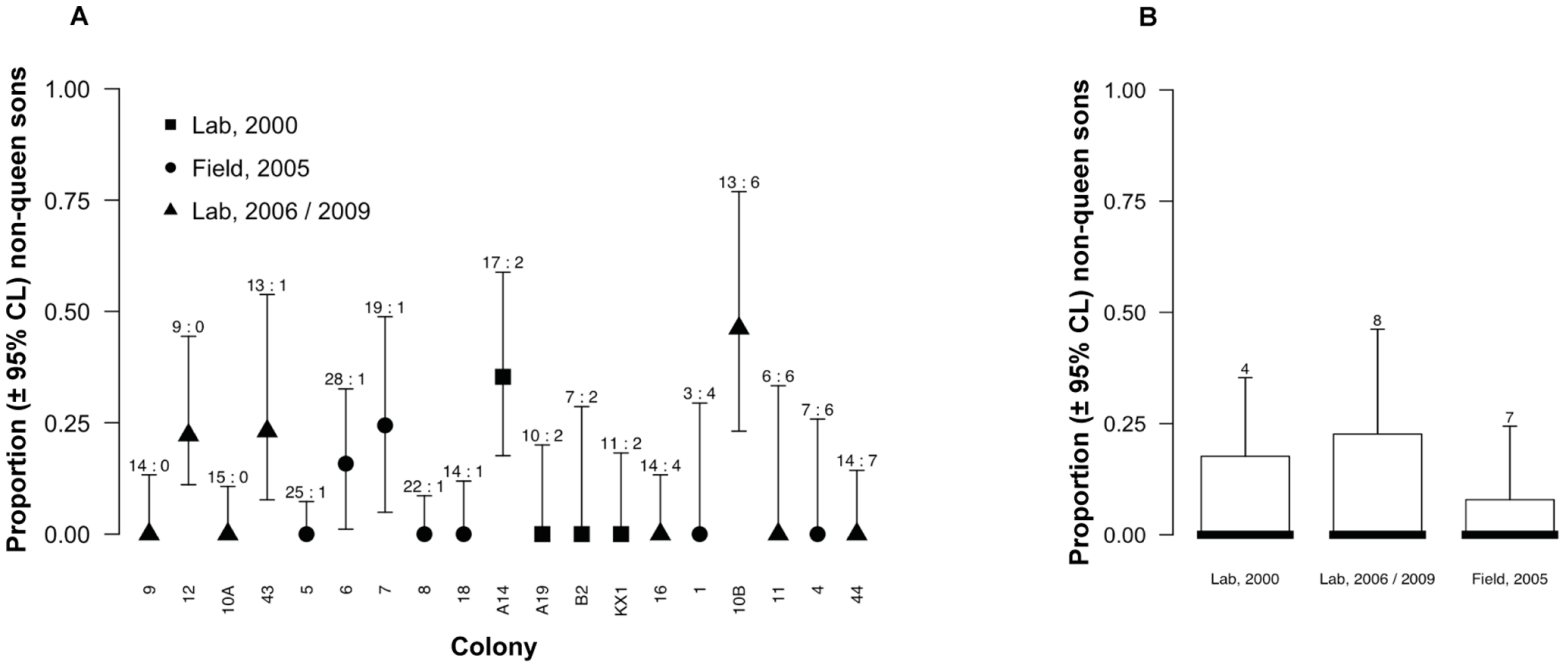
The maximum likelihood (ML) estimate of proportions of non-queen sons (i.e. intermorph and/or worker sons) among the adult males in the colony, corrected for non-detection. Laboratory samples from 2000 [11] were reanalyzed from the original data; all other data are from the present study. Error bars denote 95% confidence intervals. 1a) Individual results for the 19 genotyped colonies, sample sizes are given as number of genotyped males : numbers of intermorphs per nest. 1b) Boxplot of the ML estimates for samples from [55], field colonies collected in 2005 and lab colonies. Depicted are medians, 3^rd^ quartiles and maxima. Numbers above whiskers denote the number of genotyped colonies.

None of the males were heterozygous at either microsatellite locus, which is consistent with all of them being haploid rather than diploid males (typically infertile) such as are occasionally produced in Hymenoptera with single- or multi-locus complementary sex determination. The “Square root of number of intermorphs per colony”” was not significant (df = 18, F = 0.33, p = 0.57) in the logistic regression on the maximum likelihood estimate of the proportion of non-queen sons, indicating that the proportion of non-queen sons does not change with intermorph number when there are 1–7 intermorphs in the colony. The overall median proportion of non-queen sons among all the adult males in the genotyped colonies was 0%, but varied considerably (1^st^ quartile, 3^rd^ quartile, range; 0%, 19%, 0–46.2%) (Fig. 1a). The proportion of non-queen sons did not differ between laboratory colonies from 1998 (reanalyzed from [55]), laboratory colonies from 2006/2009, and field colonies from 2005 (df = 16, F_1, 16_ = 0.232, p = 0.80) (Fig. 1b). The total number of eggs produced after six days was significantly higher for colony fragments consisting of 10 workers and one intermorph (Median, 1^st^ quartile, 3^rd^ quartile, range; 6, 4, 18, 0–42) than for 11 workers alone (3.5, 2, 6, 0–20) (W = 269.5, p = 0.013), and significantly higher for colonies consisting of one queen and 10 workers (35, 22.5, 63, 0–75) than for colonies with intermorphs (W = 57, p<0.001), and reached a maximum after 12 days (data not shown).

Colony fragments with one intermorph and 10 workers produced ∼1.7 more eggs within 6 days than groups of 11 workers. However, the overall contribution of intermorphs to the production of non-queen sons is considered small because the colonies collected during the mating season in 2005 and 2006 with intermorphs (n = 20) had ∼130 times more workers (median, 1^st^ quartile, 3^rd^ quartile, range; 524.5, 343.5, 733.25, 117–1370) than intermorphs (4, 1.75, 6, 1–16). A negligible contribution of intermorphs to the production of adult males implies that the number of non-queen sons does not differ significantly between colonies with and without intermorphs.

### Sex ratio and sexual production is independent of intermorph number

**Figure 2:**
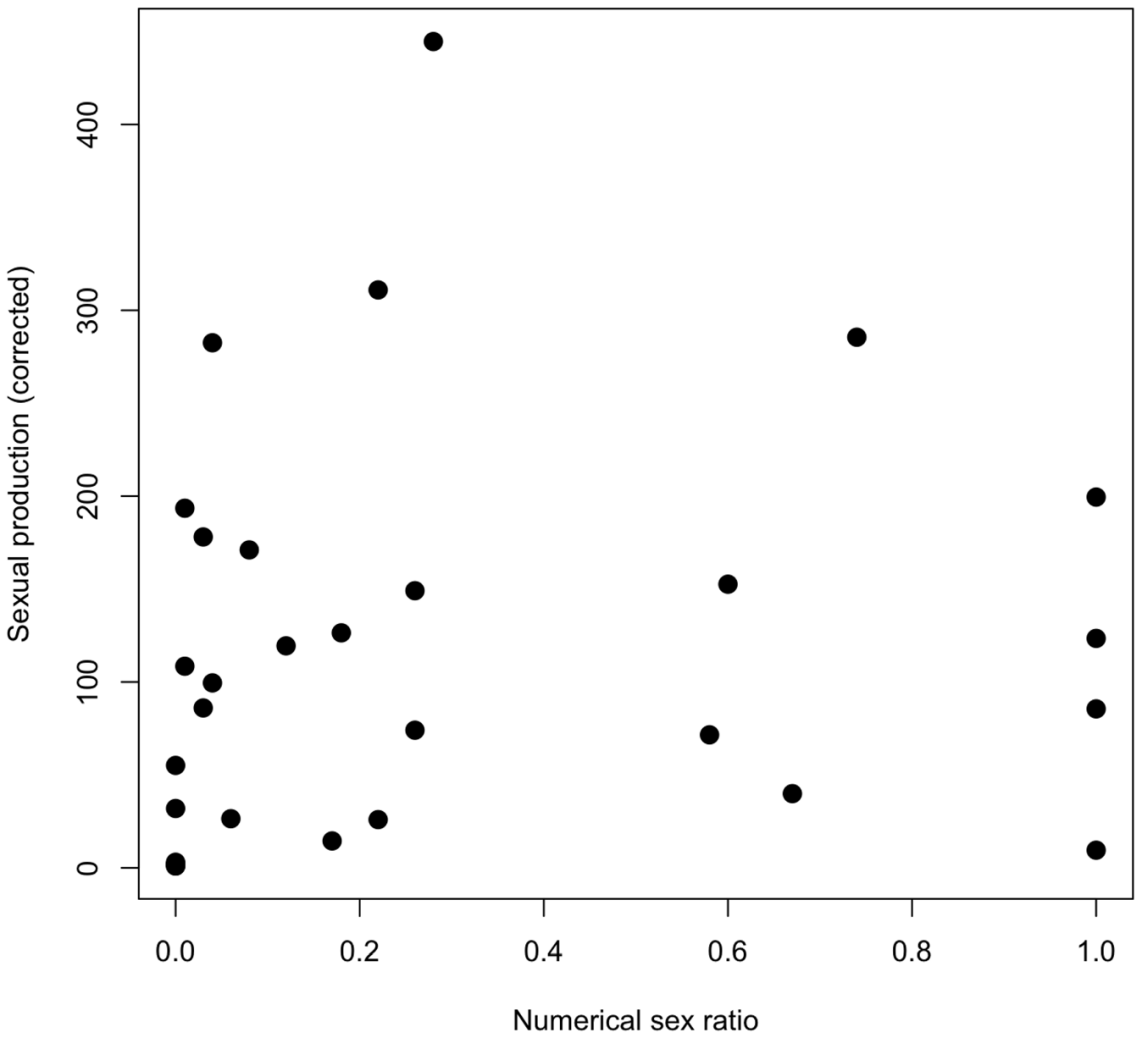
Biplot of numerical sex ratio (i.e. the proportion of virgin queens among all sexuals in the colony) and total sexual production *S_i_* (including the correction for higher investment in females) across colonies. These data show a tendency for split sex ratio strategy regardless of investment.

Like in many other eusocial Hymenoptera [22], the colony-level numerical sex ratios were strongly split, meaning that colonies tended to specialize in producing mostly mature virgin queens or mostly mature males (Fig. 2). Colonies with intermorphs did not have a significantly higher “Sexual production” than colonies without intermorphs (Wilcoxon rank sum test: W = 101.5, p = 0.73). “Sexual production” increased significantly with the number of workers per colony (GLM, df = 29, F = 5.01, p = 0.033), while “Square root of number of intermorphs per colony” did not. The analysis of the colony numerical sex ratio showed that the covariates “Number of workers per colony” and “Square root of number of intermorphs per colony” were not significant. The covariate “Sexual production” was also not significant, indicating that the colony sex ratio was independent of the total investment into sexuals.

## Discussion

Aim of our study was to determine the effect of intermorphs on the sexual production of colonies of the ant *Crematogaster smithi*. As shown previously [49], the number of intermorphs increased with colony size. Surprisingly, however, we did not detect any direct effect of intermorphs on colony reproduction.

Even though intermorphs have a higher direct fitness compared to workers their direct fitness was estimated to be surprisingly low. Even in colonies with a significant proportion of non-queen sons, workers outnumber the intermorphs by two orders of magnitude. Thus, only an estimated negligible proportion of adult males were intermorph sons, while the majority were offspring of the queen and the rest were offspring of normal workers. Hence, it is unlikely that intermorphs are a selfish caste (comparable to the “dwarf” queens in some stingless bees, [68]) that does not give any benefit to the colony. Assuming a conservative estimate of 1% intermorph sons, the life-for-life relatedness coefficient of workers to males would be only 0.5% higher in colonies with one or more intermorphs than in colonies without intermorphs (0.265 vs. 0.264, respectively). Similarly, the life-for-life relatedness of queens to males would be only 0.5% lower in colonies with than in colonies without intermorphs (0.470 vs. 0.473). At least for our sampling years we therefore have to reject the earlier hypothesis [49] that intermorphs are an “extended phenotype” of their worker sisters that helps the latter to replace brothers with more related nephews while avoiding the colony-level cost of inefficient worker reproduction.

In contrast to a previous assumption [49], feeding larvae and queens with eggs laid by intermorphs neither affected total sexual production nor sex ratio. Instead, sexual production was correlated with colony size, but resources were not necessarily allocated towards the more costly virgin queens. Most colonies produced a numerical sex ratio that was considerably more or considerably less queen-biased than the 1∶1 queen optimum (Fig. 2), strongly suggesting that workers and/or queens follow a conditional strategy for sex allocation. Numerical sex ratio was not associated with the number of intermorphs and total sexual production did not differ between colonies with and without intermorphs. This indicates that at least during the sampling years, colonies did not rely on the eggs laid by intermorphs to produce costly virgin queens or to increase the total sexual output.

Finally, we can reject a trivial proximate explanation for the occurrence of intermorphs, namely that intermorphs arise from queen-destined larvae whose development was somehow impaired (e.g., [69]). First, the number of intermorphs was not correlated with the number of virgin females. Second, the production of intermorphs and virgin queens is temporally decoupled: colonies collected in 2005 produced new intermorphs at irregular intervals throughout the year in the laboratory, but they produced sexuals from overwintering larvae one year after collection, approximately synchronously with other colonies.

Overall, our study does not give a clear explanation for the tight correlation of colony size and intermorph number. The recently documented widespread existence of intermorphic females in species of the subgenus *Orthocrema* [51]–[54] prevents us from simply dismissing intermorphs as a non-adaptive idiosyncrasy of *C. smithi*. Intermorphic females have now been described for species living in such diverse habitats as in the soil in dry juniper-oak–pinyon forests (*C. smithi*, [49]), under epiphyte mats in the canopies of montane wet forests (*C. bryophilia*, [52]), and branches on trees or on the ground in rainforests, dry forests, and spiny forests (*C. rasoherinae*, [53]). Intermorphs and the provisioning of the queen and larvae with viable eggs might be an ancestral state in this taxon that originally served to overcome food shortage under the most diverse ecological conditions and now is retained even where it has only small fitness effects.

Previous studies in polymorphic social insects have shown that it is often difficult to reconcile empirical findings with theoretical predictions about adaptive demography [70]–[76]. More information on the occurrence, ontogeny and behavior of intermorphs in other species of *Crematogaster* might be helpful to determine the proximate and ultimate causes of their existence.
